# Infections as ecosystems: community metabolic interactions in microbial pathogenesis

**DOI:** 10.1128/iai.00530-24

**Published:** 2025-08-04

**Authors:** Aanuoluwa E. Adekoya, Shannon R. West, Sydney K. Arriaga, Carolyn B. Ibberson

**Affiliations:** 1 Department of Microbiology, University of Tennessee 189504, Knoxville, Tennessee, USA; University of California at Santa Cruz, Santa Cruz, California, USA

**Keywords:** human microbiome, metabolism, microbe-microbe interaction, physiology, microbial ecology

## Abstract

Microbes rarely exist alone; instead, they live in dynamic multi-species communities with a range of metabolic capacities. To establish within a polymicrobial community, an organism must compete with the other members of the community for space and nutrients. In addition, microbes form complex metabolic interdependencies in polymicrobial environments, and these nutrient exchanges are central to overall community function. Interactions between microbial community members dictate key processes, including nutrient cycling, tolerance to disturbances, and disease progression, and these interactions are known to depend on the environment in which they are measured. Therefore, understanding these ecological interactions is fundamental to our understanding of community composition, function, and impacts on disease. In this mini-review, we will describe the mechanisms microbes use to exchange nutrients in host-associated environments, with a focus on the oral and respiratory tracts. We will particularly emphasize the environmental factors that influence community composition and how interactions between organisms, ranging from cooperation to competition, impact nutrient bioavailability and overall community function during infection.

## INTRODUCTION

Microbial communities are present on nearly every surface of the human body. These microbiomes exist as dynamic ecosystems that are influenced by heterogeneous nutritional and chemical landscapes, the ability to attach to surfaces, and interactions with the host immune system. Microbes within microbial communities exist within a metabolic economy, dictated by the sequestration and exchange of nutrients, that is central to controlling bacterial physiology, establishment within a niche, and co-existence with other community members. Furthermore, in pathogenesis, microbial metabolism is known to play a central role in virulence and the ability of pathogens to survive and persist within a host. Although studies into the mechanisms of pathogenesis have occurred for over 200 years, until only recently, these mechanistic studies have primarily focused on single organisms in isolation and have largely ignored the metabolic influence of the resident microbiota and how interactions between organisms impact disease. While this work has been foundational toward understanding how microbes cause disease, the collective role of microbiome metabolism in pathogenesis has been largely overlooked. Here, we will expand on this framework and discuss the interplay between the host environment and microbiome metabolism and the impacts on disease progression in host-associated polymicrobial communities. Although the concepts we will address are applicable to a wide range of host-associated microbiomes, for the purpose of this mini-review, we will primarily focus on interactions that occur in the oral and respiratory tracts where we have expertise. We will first discuss how the host environment influences community metabolism and disease outcomes, with an emphasis on how these environments are inherently dynamic and heterogeneous. We will then discuss microbial interactions in host-associated communities and the context-dependent nature of these interactions. Our overall goal is to provide insights about microbiome metabolism in a host and its impacts on disease progression. Increased understanding of community metabolism may allow these collective interactions to be leveraged to support more resilient microbiomes that prevent the progression of disease and improve human health.

## HOST-ASSOCIATED ENVIRONMENTS ARE DYNAMIC

**Fig 1:**
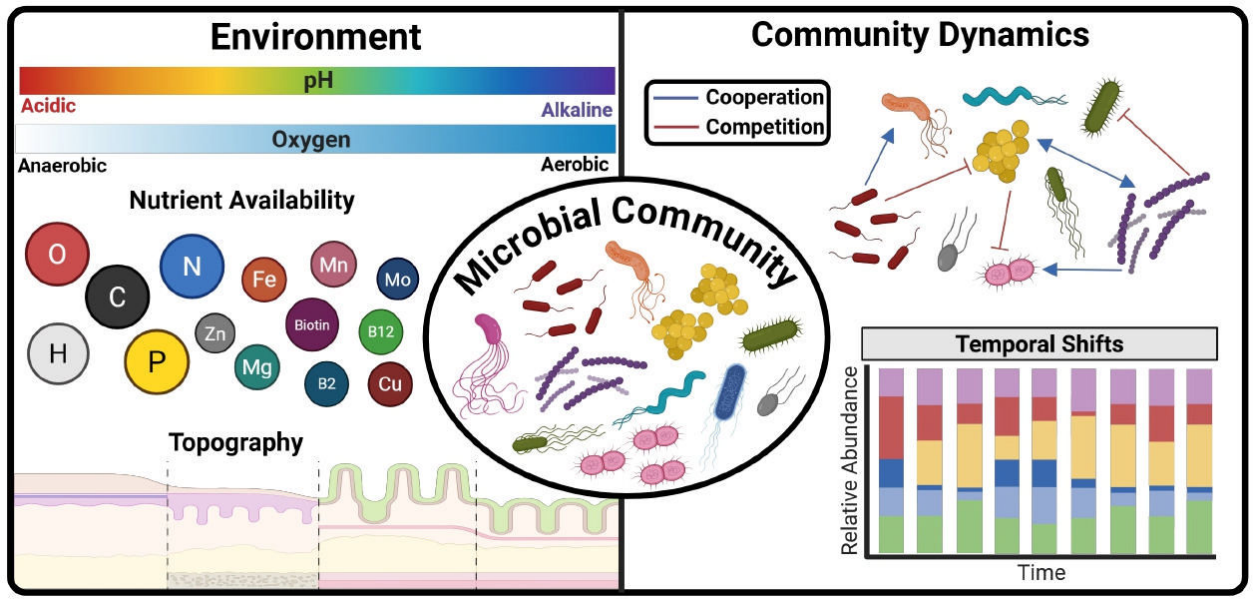
Drivers of microbial community dynamics in host-associated environments. Environmental factors, such as nutrient availability, oxygen, pH, and tissue topography, collectively shape the composition and microbiome metabolism of microbial communities within the host. The central panel represents a snapshot of a microbial community at a given time. The right panel illustrates community dynamics, including temporal shifts in microbial populations and interactions between community members that drive community composition and the microbiome metabolism. Microbial interactions are categorized as cooperative (blue) or competitive (red), reflecting the ecological forces that influence microbial stability, succession, and pathogenicity. This conceptual map emphasizes the context-dependent nature of microbial behavior and the importance of studying communities within physiologically relevant environments. Created in BioRender. Arriaga, S. (2025) https://biorender.com/o4otdmw.

Host-associated environments are dynamic and multifactorial ecosystems that require the constant adaptation and evolution of the microbial communities they harbor (1, 2). These microbiomes consist of individual populations of microbes that are specialized based on the local environment within a specific habitat within the host (e.g., periodontal cavity, lungs, large intestine, vagina, etc.) (3, 4). Microbial community composition, spatial structure, and interactions in these environments are strongly influenced by factors, such as nutrient heterogeneity, topography, and fluctuating environmental conditions like pH and oxygen levels (3). These factors select for microbial communities that are well adapted for survival in distinct micro-environments to form cohesive metabolic networks to maintain colonization throughout the host (2, 3). In this section, we will discuss how these micro-environmental factors shape overall community structure and interactions between community members (Fig. 1).

### Nutrient heterogeneity and cycling

Microorganisms require nutrients, which serve as the building blocks for the biosynthesis of cellular components and energy generation and play a central role in the regulation of key biological processes (5–7). These core macronutrients include carbon, hydrogen, nitrogen, oxygen, and phosphorus and are essential for constructing structural components, such as the cell wall, or for the biosynthesis of nucleic acids for replication (6–8). Trace nutrients, such as vitamins (9–15) and metals (16–21), also serve as cofactors for enzymatic reactions that are critical to microbial metabolism. As these nutrients are important for the growth, reproduction, and overall survival of the microbial community, nutrient availability within host-associated infections significantly influences the microbial community composition (3, 22–25). Notably, bioavailable nutrient pools in these systems are dynamic, with a constant influx and turnover of nutrients into each microenvironment. In the host environment, core essential nutrients are often sequestered in host-derived macromolecules and polymers that must be degraded or modified to be bioavailable. Furthermore, the nutrient pools are also influenced by inflammatory responses of the host, including increased vascular permeability, metabolite release from tissue breakdown, host metabolism and synthesis of macromolecules, and microbial metabolic activities to release bioavailable nutrients as sources of sugars, lipids, amino acids or other nutrients, including metals, for microbial metabolism and energy generation (26–29). This continuous release and cycling of host metabolites and bacterial metabolism supports a sustained influx of nutrients into the infection environment, providing a selective pressure for certain populations of microbes that can efficiently use these resources.

These nutrient exchanges are also influenced by temporal and spatial constraints and drive taxonomic structure and nutritional interdependencies. For example, mucosal surfaces, such as those in the respiratory tracts, are lined with epithelial cells, which secrete mucins that may serve as an energy source for resident microorganisms (30). In people with cystic fibrosis (pwCF), accumulated mucus secretions create a hypoxic and mucin-rich environment that fermenting anaerobes can use as a carbon source (26, 31–33). This fermentation of mucins leads to the release of short-chain fatty acids (SCFA) and amino acids that serve as nutrient sources for non-fermenting bacteria, such as *Pseudomonas aeruginosa* (26, 31). This nutrient cycling in the infected cystic fibrosis (CF) airway supports the growth of a diverse microbial community, and it is facilitated by continuous host secretion of mucin into the airway and the microbial metabolism of mucin and its products. Another host environment where shifts in nutritional composition impact microbial community composition is the oral cavity. The oral cavity harbors a diverse consortium of microorganisms, and rapid changes to community composition, structure, and function can occur in response to nutrient availability and metabolic byproducts of bacterial metabolism (34, 35). Saliva is a main source of nutrients in the supragingival plaque (35, 36), as it is rich in glycoproteins that can be degraded into sugar molecules and proteins by bacterial enzymes and human glycosidases (36). These host-derived sugar molecules, alongside dietary sugars, can then be accessed as nutrient sources by saccharolytic bacteria, such as *Streptococcus* spp. and *Lactobacillus* spp., and result in the efficient production of organic acids, such as lactate (36–39). This acid production lowers the pH of the supragingival plaque, enhances the demineralization of the tooth surface, and supports the colonization of acid-tolerating species (36–40). For remineralization, bacterial and host proteases can break down glycoprotein amino acids that can be catabolized to ammonia as an end product. In addition, the produced lactate through these catabolic processes can be converted to weaker acids, such as acetate and propionate, by bacteria like *Veillonella* and *Actinomyces* (36, 41, 42). This constant flushing by saliva, bacterial metabolic conversion of salivary glycoproteins to sugar and amino acids, and influx of dietary sugar provide a continuous but dynamic supply of nutrients to the microbial community. While the types of nutrient cycling and heterogeneity described here clearly influence microbiome metabolism, what remains unknown are the strategies and mechanisms organisms employ to successfully persist through these disturbances and invade into these stable metabolic networks. Also, further investigation is needed to understand how nutrient replenishment in host-associated polymicrobial interactions influences community composition and functionality and how these dynamics differ from those observed in commonly used laboratory models.

### Topography

Each host tissue provides a unique micro-environment, with topographical variations that result from anatomical structure playing a significant role in supporting distinct microbial communities. One example of a heterogeneous host environment is the lungs. Lung airways are oxygen-rich, as they supply oxygen to every other part of the body and have a mucus lining rich in nutrients, such as mucins, that can be harnessed as nutrient sources and support bacterial colonization (28, 43). However, lung airways are also subject to ciliary motion and immune surveillance by alveolar epithelial cells, macrophages, and resident dendritic cells (44–46) that create selective pressures on the microbial community that colonizes the lung. In pwCF, *P. aeruginosa* adapts to the mucus-rich and ciliated lungs by growing in microcolonies and aggregates and through upregulation of alginate synthesis genes used for biofilm formation (47–52). The oral cavity is another highly heterogeneous environment made up of distinct physical niches, such as the teeth, gums, tongue, salivary glands, and palate. Each of these areas has unique conditions—varying levels of moisture, hardness, warmth, nutrients, and salivary composition—that create a range of microhabitats for microbial colonization (53–56). For instance, moisture levels differ across surfaces, while the roof of the mouth has only a thin layer of saliva, the floor of the mouth often contains a pool of saliva (53, 57, 58), and these variations suggest that the microbial communities in these areas may vary across different sites. The Human Oral Microbiome Project (55) is a classic example of a study demonstrating how structural complexity in the oral cavity contributed directly to the microbial community composition, even in a non-disease state, with the drier and keratinized surfaces, such as the buccal mucosa, keratinized gingiva, and the hard palate having dominance of bacteria in the phylum Firmicutes, whereas non-keratinized mucosal surfaces, such as the tongue, saliva, and tonsils with softer and moist tissue, had lower abundance of Firmicutes and higher abundance of Bacteroidetes and Fusobacteriota. Additionally, the skin, which is the largest organ in the human body and is predisposed to microbial colonization, is another example of a highly heterogeneous host environment. The skin has stratified layers of connective tissues with differing depth and layers that result in temperature, oxygen, and humidity differences (59–62). One study (63) showed that these differences among skin sites within the same host were key factors that dictate the microbial community that can survive and thrive in these regions of the body. Furthermore, these topographical differences result in biochemical heterogeneity that impacts microbial communities. For example, while sebaceous glands secrete a lipid-rich sebum that supports the growth and dominance of lipase-producing bacteria, such as *Cutibacterium acnes* in the external auditory canal, the alar crease, and the occiput (64–68), moist sites, such as the nares, axillary vault, the inguinal crease, and umbilicus, are predominated by salt-tolerant *Corynebacterium* and *Staphylococcus* species (61, 65). These moist environments are rich in sweat components, such as urea and amino acids, which can be used as nitrogen sources and salt water that places osmotic stress on the bacterial community. Microbes that live in these environments, such as staphylococci, have efficient osmoregulation or osmoprotecting transport systems (69) that make them adapted to the osmotic stress that results from the production and evaporation of sweat, allowing for staphylococcal colonization in high densities (70). The heterogeneity within host environments creates distinct microenvironments that significantly influence bacterial community dynamics, as the variations that exist affect both nutrient distribution and environmental stresses. This knowledge further emphasizes the importance of studying bacterial communities within ecologically relevant contexts that accurately reflect their niche and the need to develop or quantitatively assess experimental model systems that will better mimic microbial community dynamics (71, 72).

### pH

Within host tissues, pH gradients play a crucial role in shaping the microbial communities that thrive in these environments. These pH gradients are impacted by both host and microbial secreted metabolites, creating selective pressures that influence which microorganisms are capable of surviving and dominating. For example, in the oral cavity, in non-caried individuals, the tooth surface is dominated by non-pathogenic bacteria, including *Streptococcus sanguinis*, *Streptococcus mitis*, *Streptococcus oralis*, and *Streptococcus gordonii* (37, 39, 73, 74). The presence of these commensal streptococci at a high relative abundance reduces the colonization of cariogenic (pathogenic) streptococci and other acid-producing or acid-tolerating bacterial species (37). However, streptococci have efficient carbohydrate metabolizing machinery (37), which leads to the release of acids. As a result, the acidification of the teeth below a critical point allows the relative abundance of cariogenic bacteria, such as *Streptococcus mutans* and *Lactobacillus salivarius* to increase (37–40). Dietary habits, which include consumption of foods rich in fermentable carbohydrates, in turn, expose the oral cavity to constant acidification by acid-producing streptococci, allowing cariogenic bacteria to thrive in dental caries (37, 38, 73). The vaginal cavity is another host tissue with strong selective pressures from pH fluctuations. The normal vaginal environment is maintained at an acidic pH (3.8 to 4.5) largely due to lactic acid production by *Lactobacillus* species (75). These lactobacilli species dominate the vaginal microbiota, and their acid production prevents colonization by pathogens, which are usually non-acid-tolerant (75–77). However, alteration of the vaginal tract pH by various host factors, including hormonal changes or sexual activities, as well as microbial metabolism, occasionally results in pH level increases, shifting the environment to a more neutral or alkaline state. Such pH fluctuations in the host environment can create conditions that can lead to dysbiosis, allowing the growth of opportunistic pathogens. These shifts in microbial community composition driven by pH fluctuations resulting from microbial metabolism highlight how microbial metabolic processes can induce ecological changes within the infection environment. This, in turn, shapes the community composition and plays a significant role in disease establishment and progression.

### Oxygen

As a preferred electron acceptor in many metabolic processes, oxygen availability directly influences microbial metabolism, community interactions, microbial survival, and overall disease progression (78–84). Variations in oxygen levels across host tissues, which are driven by factors, such as blood flow, inflammation, and recruitment of immune cells to infection sites, create unique niches that shape microbial community structure and function. Surface-exposed tissues, such as the nasal epithelium and the lungs, are oxygen-rich. However, specific conditions can lead to localized oxygen depletion. For example, in conditions, such as chronic obstructive pulmonary disease (COPD), the deeper parts of the airways, including the alveoli, may be constricted due to inflammation and mucus buildup, resulting in lower oxygen levels or alveolar hypoxia (85–88). Similarly, in pwCF, impaired mucus clearance leads to the accumulation of a dense mucus layer in the airways, creating localized oxygen gradients as well as impacting microbial community dynamics (89). Specifically, Silveira et al. (90) showed that in pwCF, an oxygen shift coupled with nutrient availability drives the stability of the microbial community (90). Colonization by fermenting facultative and obligate anaerobes, such as *Streptococcus*, *Veillonella*, and *Prevotella* spp., allows the efficient degradation of mucin to release free amino acids and short-chain fatty acids (26, 90). These nutrients support the growth of fast-growing organisms, such as *P. aeruginosa* (26, 90), which, in turn, uses up the oxygen within the mucus layer, creating a hypoxic environment and facilitating the growth of obligate anaerobes, such as *Veillonella* and *Prevotella* spp. (90). Moreover, this oxygen limitation can also alter virulence factor production. For example, *P. aeruginosa* produces phenazines under hypoxic conditions, which enhance its survival and contribute to host tissue damage (91, 92).

Similarly, oxygen gradients across the skin play a significant role in modulating the microbial community composition and functional dynamics of these environments. The skin surface is generally well-oxygenated due to direct atmospheric exposure and robust vascularity (93). Commensals, such as *Staphylococcus epidermidis* and *Cutibacterium acnes*, typically inhabit these dry and exposed surfaces (94). However, tissue injury and wound healing processes initiate a dynamic oxygen gradient characterized by spatial and temporal variations (95, 96). Initial wound stages maintain high oxygen levels, which support colonization by facultative anaerobes, such as *Staphylococcus aureus* and *P. aeruginosa*. However, as wound infection progresses, hypoxic microenvironments are created due to reduced blood flow, high inflammatory responses, and increased metabolic activity of aerobes and facultative anaerobes (97–99). This, in turn, triggers metabolic shifts that facilitate the formation of hypoxia and foster an environment that is conducive to anaerobic bacteria (78). Several studies have shown that chronic wounds harbor a diverse microbial community rich in anaerobic species, such as *Anaerococcus*, *Finegoldia*, and *Peptoniphilus* (79, 100–102). There is also oxygen-dependent zonation, with the center of the wounds exhibiting higher levels of hypoxia than the margins. This creates distinct niches and leads to spatial segregation of these communities, with aerobes predominantly colonizing the oxygenated margins and facultative or obligate anaerobes segregating toward the center (103, 104). As oxygen is required for wound healing to support the enzymatic processes and immune response (105), the diminished levels of oxygen in the wound environment contribute to delayed healing and support the persistence of the microbial community.

Overall, the host environment presents diverse microenvironmental conditions that create selective pressures and metabolic bottlenecks, favoring certain bacterial species while limiting others. As these species are distinct in their metabolic capacities, microbial community metabolism is, in turn, directly impacted by these changes in community composition. Furthermore, within these dynamic conditions, the bacterial species exhibit adaptive physiological responses and engage in various interspecies interactions—ranging from competitive to cooperative—that ultimately shape the microbial community composition, function, and overall disease progression. These interactions between microbial species and their impacts on microbiome metabolism and disease progression will be the subject of the next section of this review.

## SPECTRUM OF MICROBIAL INTERACTIONS

**Fig 2:**
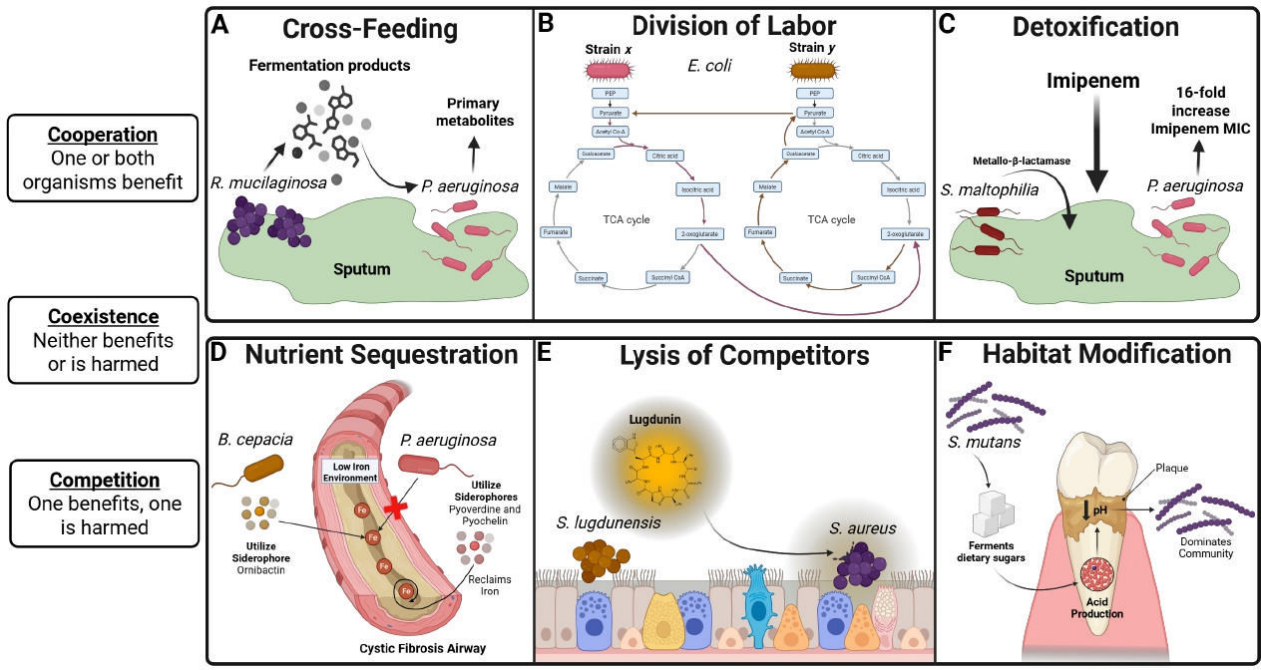
Conceptual model of microbial interactions in polymicrobial communities. Microbes interact through a range of ecological strategies that shape community structure and infection outcomes, ranging from cooperation to co-existence to competition. Here, we illustrate three cooperative mechanisms (top row) and three competitive mechanisms (bottom row) organized by interaction type across this spectrum. (**A**) Cross-feeding: *R. mucilaginosa* produces fermentation byproducts, such as lactate, which are utilized by *P. aeruginosa* to produce primary metabolites, such as glutamate (120). (**B**) Division of labor: subpopulations of *E. coli* (strain x and strain *y*) partition the tricarboxylic acid (TCA) cycle, exchanging 2-oxoglutarate and pyruvate. This metabolic division reduces proteomic burden and supports shared growth (121). (**C**) Detoxification: *S. maltophilia* expresses metallo-β-lactamase to inactivate the antibiotic imipenem, providing exposure protection to other community members, including *P. aeruginosa*, in polymicrobial infections (111). (**D**) Nutrient sequestration: in the cystic fibrosis airway, *B. cepacia* and *P. aeruginosa* secrete distinct siderophores (ornibactin, pyoverdine, pyochelin) to outcompete one another for limited iron (122). (**E**) Lysis of competitors: *S. lugdunensis* secretes lugdunin, an antimicrobial fibupeptide, causing lysis of neighboring cells, such as *S. aureus* (123). (**F**) Habitat modification: *S. mutans* ferments sugars, producing acid that lowers local pH in the oral cavity. This selective environment excludes less acid-tolerant species and allows *S. mutans* to dominate plaque communities (40). Together, these cooperative and competitive strategies impact microbial community stability and resilience. Created in BioRender. Arriaga, S. (2025) https://BioRender.com/h4d9d57.

Microbial interactions are an inherent part of microbial communities (106), and, in this review, we broadly categorize microbial interactions along a spectrum ranging from cooperation to coexistence to competition (Fig. 2). In recent years, research has provided insight about the diverse and context-specific interactions, which occur between commensal microbes, pathogens, and hosts (23, 107, 108). More specifically, studies have demonstrated that metabolic interactions between microbes within a polymicrobial infection can contribute to microbial community function, increased virulence of pathogens, host health, and disease progression (109–113). However, most studies of microbial metabolic interplay during infection have focused on two microbes or a single microbe and host. While these studies have given fundamental insights, this approach has largely ignored the combined influence of metabolic interactions between the commensal microbes, pathogens, and host at an infection site. Over the past two decades, emerging and new technologies in the field of microbial ecology have led to the discovery of key characteristics of microbial communities across different habitats. For example, an analysis by Machado et al. of the Earth Microbiome Project data set (114) containing microbiomes ranging from free-living (aquatic and soil environments) to host-associated (animals and plants) showed that species co-occurrence across microbial communities has a distinct polarized pattern of either cooperative or competitive as the number of species increases above three in a community (115). Furthermore, the authors found that cooperative communities are present in both free-living and host-associated habitats, while competitive communities are primarily found in soils (115). This study demonstrated that different environments select for distinct communities, and that host-associated communities tend to be cooperative. Some key characteristics of cooperative communities are metabolic dissimilarity, lower nutrient requirement overlap, and multiple complementary auxotrophies, which lead to increased cross-feeding (115, 116). Additionally, species within cooperative communities tend to have smaller genomes most likely due to adaptive metabolic gene loss (115). Conversely, microbes within competitive communities have larger genomes with more competitive capabilities, such as increased metabolic and antimicrobial activity genes (115). Consistent with the need for competitive mechanisms of interaction, species co-occurring within competitive communities are generally metabolically similar and interestingly share close phylogenetic relatedness (115). Beyond community structure, mathematical simulations of microbes in communities demonstrate that, within a given habitat, there is a dynamic hierarchical structure to metabolic strategies within the community based on the metabolite preferences of each participating microbe (117). This metabolic adaptability may be a major contributor to the development of cooperative or competitive phenotypes in microbial communities. Importantly, the cooperative and competitive mechanisms utilized by neighboring microbes in host-associated infection environments may impact the local nutrient landscape, further contributing to community structure and stability. Although there is evidence that host-associated microbial communities exhibit cooperative or competitive metabolism that supports their stability over time (115, 118, 119), questions remain about the specific interactions within the entire metabolic network that contribute to these patterns. In the following sections, we will discuss the range of metabolic interactions that occur between microbes within the host and the impacts these interactions have on community function and disease.

### Cooperation

Cooperation can be defined as beneficial interactions of and between microbes, such as exchanging byproducts or sharing metabolic tasks, that lead to mutual persistence (124). These interactions allow microbes to live in a community together and perform biochemical processes that would be impossible or inefficient in isolation. Additionally, cooperative metabolic interactions synchronize resource use and enhance the survival of the community members. However, this dependence on metabolic cooperation can also make the community more susceptible to invasion by highly fit competing microbes, which can disrupt the community structure and allow the invading microbe to dominate (115). In terms of host-associated polymicrobial infections, metabolic cooperation contributes to pathogenesis and disease progression (110, 120, 125, 126). For example, the development of dental diseases, such as periodontitis, is associated with a pathogenic bacterial consortia known as the ‘red complex’ (*Porphyromonas gingivalis*, *Treponema denticola*, and *Tannerella forsythia*) (126). Genomic analysis of the ‘red complex’ showed that cooperative biosynthesis and production of fatty acids, particularly butyric acid, are an essential metabolic pathway for these species to persist and cause inflammation (127). Cooperative mechanisms that lead to mutual persistence within a microbial community include inter- and intra-species interactions, such as cross-feeding, division of labor, and detoxification (Fig. 2A through C). Here, we describe these strategies in more depth and discuss the role of cooperation in polymicrobial infections.

#### Cross-feeding

Cross-feeding occurs when a microbe utilizes a metabolite produced by another microbe. Polymicrobial cross-feeding has been shown to be required for colonization, persistence, and virulence of pathogens in coinfections (110, 112, 127). Many cross-feeding mechanisms that have been identified to date occur through the secretion of waste products as shared goods (26, 120). For example, metabolic waste products secreted by a consortia of anaerobic bacteria isolated from CF sputum allowed *S. aureus* to gain nearly a fivefold growth increase compared to a control lacking the anaerobic consortia (26). Similarly, when cross-fed with ^13^C-labeled supernatant from *Rothia mucilaginosa* grown in artificial sputum media, *P. aeruginosa* generated the primary metabolite glutamate using *R. mucilaginosa*-secreted fermentation products (120). This is an important cross-feeding reaction because glutamate is a major cell wall component of *P. aeruginosa*, and access to increased glutamate during iron limitation has been shown to increase the growth yield of exotoxin A-producing *P. aeruginosa* (128). Both studies demonstrate the ability of pathogens to cross-feed metabolic products secreted by commensal bacteria in CF infection, potentially aiding in the persistence and virulence of the pathogens in this chronic infection. However, cross-feeding interactions are not limited to interactions in CF. As an example of bacterial cross-feeding in another environment, ornithine is a key metabolite cross-fed from *Enterococcus faecalis* to *Clostridiodes difficile* within the gut microbiome (110). This re-shaping of the metabolic environment through the provision of ornithine, in turn, cues the depletion of arginine and has been shown to increase the overall fitness and persistence of *C. difficile* in the gut, triggering toxin production (129). Timme et al. (2024) also described an example of how the metabolism of acetoin provides a strong link between clinical strains of *A. baumannii* with *S. aureus. S. aureus* produces acetoin to maintain pH homeostasis under certain conditions, including oxygen-limited environments (130). Interestingly, *A. baumannii* utilized and rapidly depleted *S. aureus*-produced acetoin when grown solely in cell-free supernatant of overnight *S. aureus* culture to support its own growth and bypass the cell density reduction effect of the phenol-soluble modulins secreted by *S. aureus* (131). Together, these examples clearly demonstrate how metabolic cross-feeding can have significant impacts on pathogenesis, resulting in synergies which increase burdens of pathogens.

In addition to cross-feeding via secreted metabolites as described above, there are also rarer examples of cross-feeding that occur through direct contact between microbes. One interesting example was engineered by Pande et al. using auxotroph mutants of *Escherichia coli* and *Acinetobacter baylyi* grown in chemically defined minimal media. These auxotrophic mutant strains of *E. coli* and *A. baylyi* reciprocally exchanged amino acids through intercellular nanotubes produced by *E. coli* (132). Not surprisingly, the physical separation of each species eliminated cross-feeding in this case, which also highlights the role of spatial structure in polymicrobial communities and highlights an interesting point that pathogens cannot access the benefits of cooperative metabolism when they are spatially separated from communities (133). While to date this novel mechanism of interaction has only been observed *in vitro* with more evidence from the soil bacterium *Bacillus subtilis* (132, 134–136), it highlights the diverse range of mechanisms microbes use to interact, and we anticipate additional novel interaction mechanisms to be identified in host-associated communities as the field and technologies continue to develop. Collectively, these elegant cross-feeding mechanisms identified through pairwise interactions have furthered our knowledge of nutrient exchange within polymicrobial communities. However, the signals that drive metabolite exchange and how these nutrients are shared in more complex communities remain open questions.

#### Division of labor

Division of labor (DOL) occurs when populations within a community perform different steps in a metabolic pathway, consequently reducing the metabolic or proteomic burden of each population (121, 137). As an example of intraspecies DOL in the context of a polymicrobial infection, Qin et al. (138) showed that amino acid auxotrophism could simulate DOL among clinical isolates of *P. aeruginosa* from pwCF. In this study, the authors demonstrated that strains auxotrophic for methionine and strains auxotrophic for arginine could provide growth complementation for each other, which directly supports *P. aeruginosa’*s evasion of host nitric oxide and increases pathogen burden (138). There has also been evidence of non-nutritional DOL between *P. aeruginosa* strains. For example, mucoid and non-mucoid strains of *P. aeruginosa* can shield their populations from host immune responses in a bidirectional manner (139). The mucoidal strains of *P. aeruginosa* produce alginate as part of their extracellular polymeric substance (EPS) that shields the *P. aeruginosa* community from the toxic effect of the host immune peptide, LL-37. Furthermore, the non-mucoid strains are efficient in catalase synthesis that protects the community from the toxic effect of hydrogen peroxide. There has also been an *in silico* simulation of DOL in metabolic networks, which showed that when under constraint, two distinct subpopulations of *E. coli* formed a metabolic strategy to perform different parts of the TCA cycle through the exchange of the TCA cycle intermediate 2-oxoglutarate and the glycolytic intermediate pyruvate (121). This demonstrates how DOL could develop in an isogenic population, potentially diminishing the metabolic burden of both populations. Additionally, a study by Voelz et al. described an example of DOL among two pathogenic fungal subpopulations where *Cryptococcus gatti* persisted intracellularly in leukocytes by a subpopulation adopting tubular mitochondria, which not only provided protection against ROS but also allowed neighboring cells to rapidly proliferate, causing increased pathogen burden (140). It is important to note that interspecies DOL in polymicrobial communities can be challenging to study because of the many factors affecting the metabolism of each population, including the sharing of metabolites within the community (137, 141). However, a recent modeling study indicated that partial metabolism in microbial communities could occur through DOL that maximizes proteome efficiency, increasing the fitness of the microbial community members (137). DOL is a critical cooperative strategy that has been shown to be useful for the maintenance of microbes of the same species, and we anticipate it may also serve as a strategy for the collective survival of distinct microbes. Hence, understanding DOL in host-associated commensal communities may provide insight on areas of weakness that pathogens can target for invasion into and persistence within the metabolic economy of microbial communities.

#### Detoxification

Detoxification is considered the reduction or removal of harmful products from a system or a particular environment. Bacteria have evolved many detoxification mechanisms for survival, such as transformation of harmful toxins, reduction of destructive reactive oxygen species (ROS), and production of antibiotic-inactivating enzymes (111, 142, 143). One underexplored area of research is how detoxification contributes to shaping the microbial community at infection sites. A recent study showed that some environmental and pathogenic bacteria can chemically modify 2-heptyl-4-hydroxyquinoline N-oxide (HQNO) produced by *P. aeruginosa* to less toxic forms (142). This detoxification of HQNO demonstrated the ability of certain bacteria to detoxify a potent toxin and virulence factor produced by a pathogen, which could benefit other microbes found in the same community. Additionally, commensal microbes can protect pathogens from ROS produced from host immune defenses (143). For example, *in vitro*, *S. aureus* was protected from killing by ROS when augmented with heat-killed *Micrococcus luteus*. The resulting increased survival of *S. aureus* was due to the *M. luteus-*produced catalase detoxifying the environment of ROS. In addition to the mechanisms described above, antibiotic deactivation by one pathogen can act as a shared benefit to a coinfecting pathogen (111). For example, *P. aeruginosa* was shown to benefit from *Stenotrophomonas maltophila* producing a metallo-β-lactamase, which inactivates carbapenems, such as imipenem and meropenem (111). The production of this antibiotic-inactivating enzyme by *S. maltophila* consequently increased the imipenem tolerance of *P. aeruginosa* by 16-fold (111), highlighting the importance of considering the entire microbial community when selecting treatments for infections. Similarly, there is additional evidence that β-lactamase-producing bacteria that are part of the normal pharyngeal microbiota can decrease the effectiveness of penicillin, a β-lactam antibiotic, against Group A *Streptococcus* infections, potentially contributing to treatment failure (144–148). These mechanisms of detoxification performed by either commensal or pathogenic bacteria can act as shared goods within microbial communities, allowing pathogens to persist despite immune system defenses and the use of therapies deemed clinically effective.

### Coexistence

Microbial coexistence is defined as the ability of different microbes to occupy the same environment over time and is considered a neutral interaction where neither microbe benefits or is harmed (149). Although many interaction mechanisms modulate coexistence, microbial ecologists generally consider metabolism to be the foundation of microbial coexistence. This metabolic view of coexistence encompasses the physiological capabilities of individual populations or members within the community as well as spatiotemporal constraints. In general, we think of stable microbial communities forming cohesive metabolic networks with varying degrees of metabolic overlap and dependencies (115). Microbes can form close associations with hosts and other microbes, which can lead to auxotrophies following prolonged associations (150). Therefore, patterns of species co-occurrence in complex microbial communities can be partially explained by differing auxotrophic needs. These auxotrophies consequently form metabolic dependencies within the community metabolic network. Many examples of this shared metabolism and the pressures that drive it within microbial communities are presented in studies of the human gastrointestinal tract (107, 108, 151). However, much less is known about polymicrobial metabolic interplay in other host-associated sites, especially during infection. Theoretically, this metabolic interplay reaches equilibrium in a steady state. Yet, polymicrobial communities are rarely static over time and instead respond to the changing environment, such as an influx of nutrients, as described above, or by disturbances, such as antibiotic perturbation.

Contemporary research of polymicrobial infection ecology has used metabolite interplay as a means of explaining the coexistence of major coinfecting pathogens. This metabolic adaptability and interplay, in theory, allows for multiple pathogens to coexist in infection. However, we know that microbial physiology can differ based on microbial community composition. This is because bacteria can sense their surroundings, other microbes, and metabolites and adjust their metabolism and physiology accordingly. Hence, it is challenging to experimentally disentangle microbial coexistence from cooperation, as these metabolite exchanges frequently result in mutual or unilateral benefits. To better treat polymicrobial infections, it is crucial to overcome this limitation and understand the specific metabolic interplay and physiological responses, which underpin microbial coexistence dynamics in host-associated polymicrobial communities.

### Competition

In host-associated polymicrobial environments with limited nutrients and key resources that are essential for the growth of the microbial community, the members of the community employ various mechanisms to obtain resources and establish their dominance. These strategies are often competitive, aimed at the exclusion of other members, and can be direct, such as the synthesis of metabolites for sequestering limited nutrients or lysis of competitors, or indirect strategies, such as the manipulation of the host environment to make it unfit for other community members (119, 152–155). The outcome of these competitive interactions, in turn, shapes the microbial community structure, function, and metabolism, impacting the overall community dynamics (115, 119, 156, 157).

#### Nutrient sequestration

One key factor that pushes microbial interactions toward competition is the limitation of key resources (158), such as iron. Iron is an important trace element for many metabolic processes (16, 159); however, the host uses nutritional immunity with the production of numerous iron-binding proteins, such as heme, hemoglobin, ferritin, lactoferrin, and transferrin, to limit microbial access to free iron (20, 21, 160, 161). In polymicrobial infection environments, this iron becomes scarce to the microbial community, and microbes employ iron-scavenging mechanisms, which allow them to outcompete other community members with limited iron-scavenging abilities (162). In the airways of pwCF, the mucin-rich environment supports the growth of a diverse microbial community (26, 122). However, free iron is limited in this environment, and the bacterial community must effectively compete for iron, influencing the diversity, structure, and function of the community. For example, *Burkholderia cepacia* can sequester iron with its siderophore, ornibactin, to limit iron availability to community members that cannot access ornibactin-bound iron, such as *P. aeruginosa* (122). *P. aeruginosa*, in turn, uses its own siderophores, including pyoverdine and pyochelin, to overcome this challenge and obtain iron from this iron-limited environment (162, 163). This exploitative mechanism not only provides *P. aeruginosa* with iron for its metabolism, but these high-affinity siderophores also further limit available iron to other community members. In a similar mechanism, Bronzyna et al. showed evidence that non-siderophore-producing bacterial species can "steal" siderophores from their producing neighbors to enhance their own fitness. Specifically, *Staphylococcus lugdunensis*, which is unable to synthesize siderophores, exhibited enhanced growth under iron-limited conditions when co-cultured with staphyloferrin-producing *S. aureus* by scavenging the staphyloferrin A and B produced by *S. aureus* (164). This phenomenon was also described in the work of Zhao et al. (165). Additionally, using a *∆sfa∆sbn* double mutant of *S. aureus* (unable to produce staphyloferrins), Zhao et al. further demonstrated that *Mammaliicoccus sciuri* (formerly *Staphylococcus sciuri*) synthesizes a siderophore that is inaccessible to *S. aureus* (165). However, when co-cultured with wild-type *S. aureus*, *M. sciuri* is capable of also consuming staphyloferrin B produced by *S. aureus* while still producing its own siderophore, thereby further reducing the growth of *S. aureus* (165)*.* As many bacterial species share overlapping resource requirements, nutrient-driven competition is inevitable in polymicrobial infections. Therefore, it is important to understand how the microbial community persists and drives disease progression, even in the face of competition and nutrient limitation.

#### Lysis of competitors

Another competitive mechanism that shapes microbial community composition is bacterial interference through the production of certain metabolites, which are inhibitory to the survival of other bacterial species (166). These inhibitory strategies may be contact-dependent, requiring cell-to-cell contact for effective delivery, or contact-independent, inhibiting the growth of physically separated bacteria. In host-associated environments, many species of bacteria produce toxins, secondary metabolites, or antimicrobial peptides, which can suppress or inhibit the growth of competitors and alter the structure of the microbial community in a contact-independent manner. For example, in pwCF, the iron-sequestering advantage of *P. aeruginosa* further allows the proliferation of *P. aeruginosa* and the release of toxic products, such as 2-heptyl-4-hydroxyquinoline N-oxide (HQNO) or pyocyanin, which can kill competing *S. aureus* (122, 167, 168). As another example, in the nasal cavity, the commensal *S. lugdunensis* produces lugdunin, an antimicrobial that is effective against *S. aureus* and reduces *S. aureus* nasal colonization (123). Furthermore, *Corynebacterium accolens* was shown to inhibit the growth and nasal colonization of *Streptococcus pneumoniae* through the release of oleic acid from the hydrolysis of triacylglycerols (169).

In addition to contact-independent mechanisms via the secreted metabolites described above, some microbes have been shown to use protein secretion apparatuses for the direct delivery of toxins into neighboring cells. Several bacterial species have been shown to use secretion systems, including Type I (T1SS), Type IV (T4SS), Type VI (T6SS), and Type VII (T7SS), for cell-to-cell toxin delivery to inhibit the growth of competitive bacteria. For example, *P. aeruginosa* uses its T6SS to deliver Tse1 and Tse3, two effector proteins, to recipient *Pseudomonas putida* cells, leading to the hydrolysis of the cell wall of *Pseudomonas putida* and providing *P. aeruginosa* a fitness advantage (170). This is not limited to Gram-negative bacteria, as a recent study by Ulhuq et al. (171) showed that T7SS provided a competitive advantage to the *S. aureus* strain COL during intraspecies competition in a Zebrafish hindbrain ventricle infection model (171). Specifically, they showed that *S. aureus* strain COL uses its T7SS to deliver a toxin, TspA, to the cells of *S. aureus* strain RN6390, triggering the cytoplasmic membrane depolarization of *S. aureus* strain RN6390 and resulting in growth inhibition (171). Taken together, these competitive mechanisms used by microorganisms to gain fitness advantages during infection highlight the complexity of microbial warfare in shaping community dynamics. Understanding the intricacies of these competitive processes will provide valuable insights into the ecological strategies that persistent populations employ to outcompete other community members and dominate in host-associated environments.

#### Habitat modification

Additionally, the alteration of the host environment may provide certain bacteria with a competitive edge to exclude other members of the community. For example, in dental plaques, saccharolytic *S. mutans* efficiently ferment dietary sugars in the host that lead to the production of acids (37, 39). This acidification significantly lowers the pH of the dental biofilm, allowing the growth and domination of *S. mutans* in the plaque and inhibiting the growth of less acid-tolerant commensal bacteria, such as *S. mitis* or *S. salivarius* (37–40). This drives a shift in the microbial composition from a commensal-dominating environment to a pathogen-dominating environment. Similarly, environmental modulation through the production of acid may allow bacterial communities to limit colonization by pathogens. As seen in the glycogen-rich vaginal tract, lactic acid produced by commensal *Lactobacillus* spp. as a product of carbohydrate metabolism lowers the pH of the environment (75–77). This acidic environment provides a competitive advantage to *Lactobacillus* spp. from invasion and domination by less-acid tolerant bacteria, such as *E. coli*, *Candida albicans*, and *Gardnerella vaginalis* while conferring protection on the host (76). The diverse competitive strategies used by microorganisms highlight the dynamic and competitive nature of polymicrobial communities within a host. Importantly, these competitive mechanisms are not merely survival strategies but critical determinants that shape community structure and functional dynamics, including virulence and host-microbe interactions (172).

## TAKE AWAYS AND OPEN QUESTIONS

Microbial community metabolism is a collective activity that influences key processes, including exploitation of a host’s resources, nutrient availability, tolerance to disturbances, and disease progression, and is strongly influenced by both community composition and the host microenvironment. Despite its importance, the role of microbiome metabolism has been underexplored in pathogenesis. Here, we provide an overview of how the host environment and microbial community dynamics influence community composition, interactions, metabolism, and virulence. However, many open questions remain, particularly in host-associated microbial communities beyond the gut, including: (i) how community metabolism impacts disease progression in diverse host environments; (ii) how nutrients are exchanged in host-associated communities; and (iii) what are the dynamics of these communities before, during, and after infection. To address these questions and more, technical innovations are needed, including: (i) better model systems that more accurately mimic the *in vivo* dynamics and complexity of polymicrobial infections; (ii) temporal data sets from host-associated communities beyond the gut microbiome so community dynamics, succession, and response to disturbances, such as infection and antibiotic treatment, can be evaluated; and (iii) improved analysis models for microbiome metabolism and microbe-microbe interactions that move beyond single organisms and theoretical networks to take into account the context-dependent nature of these systems. Improving our understanding of microbiome metabolism in infection may allow us to harness microbial interactions and engineer more resilient communities that limit host damage and disease, ultimately improving human health.
